# Crop management system and carrot genotype affect endophyte composition and *Alternaria dauci* suppression

**DOI:** 10.1371/journal.pone.0233783

**Published:** 2020-06-04

**Authors:** Sahar Abdelrazek, Philipp Simon, Micaela Colley, Tesfaye Mengiste, Lori Hoagland

**Affiliations:** 1 Department of Horticulture and Landscape Architecture, Purdue University, West Lafayette, Indiana, United State of America; 2 USDA-ARS Agriculture Research Service, Madison, Wisconsin, United States of America; 3 Organic Seed Alliance, Port Townsend, Washington, United States of America; 4 Department of Botany and Plant Pathology, Purdue University, West Lafayette, Indiana, United States of America; Universita degli Studi di Pisa, ITALY

## Abstract

Managing pests in carrot production is challenging. Endophytic microbes have been demonstrated to improve the health and productivity of many crops, but factors affecting endophyte dynamics in carrot is still not well understood. The goal of this study was to determine how crop management system and carrot genotype interact to affect the composition and potential of endophytes to mitigate disease caused by *Alternaria dauci*, an important carrot pathogen. Twenty-eight unique isolates were collected from the taproots of nine diverse genotypes of carrot grown in a long-term trial comparing organic and conventional management. Antagonistic activity was quantified using an *in vitro* assay, and potential for individual isolates to mitigate disease was evaluated in greenhouse trials using two carrot cultivars. Results confirm that carrot taproots are colonized by an abundant and diverse assortment of bacteria and fungi representing at least distinct 13 genera. Soils in the organic system had greater total organic matter, microbial biomass and activity than the conventional system and endophyte composition in taproots grown in this system were more abundant and diverse, and had greater antagonistic activity. Carrot genotype also affected endophyte abundance as well as potential for individual isolates to affect seed germination, seedling growth and tolerance to *A*. *dauci*. The benefits of endophytes on carrot growth were greatest when plants were subject to *A*. *dauci* stress, highlighting the importance of environmental conditions in the functional role of endophytes. Results of this study provide evidence that endophytes can play an important role in improving carrot performance and mediating resistance to *A*. *dauci*, and it may someday be possible to select for these beneficial plant-microbial relationships in carrot breeding programs. Implementing soil-building practices commonly used in organic farming systems has potential to promote these beneficial relationships and improve the health and productivity of carrot crops.

## Introduction

Carrot (*Daucus carota* subsp. “sativus”) is an important world vegetable crop providing a significant source of beta-carotene, vitamins C and K to the human diet [1]. Carrots can be grown under a wide range of soil and climatic conditions, though carrot production is notoriously difficult due to many biotic and abiotic stress factors that can negatively affect the productivity and quality of its edible taproots. For example, Alternaria leaf blight, caused by the pathogen *Alternaria dauci*, is widely recognized as one of the most common and destructive carrot diseases [2–6]. The primary challenge associated with Alternaria leaf blight is dramatic decay of aboveground foliage making mechanical harvest of carrots difficult. This pathogen can also cause taproot decay, which reduces carrot stands as well as the quality and marketability of taproots [5–7]. Herbicides and pesticides are available to help overcome these challenges, though many growers, particularly in developing countries, lack access to these agrochemical inputs. In addition, some growers choose not to use these products due to demand for carrots grown using organic management practices. In the U.S. alone, carrots now hold the greatest market share of all crops in the organic sector, with approximately 14% of the entire crop grown using organic production practices [8]. Consequently, alternative approaches to manage pests and improve the health and quality of carrot crops are needed.

Recently, the plant microbiome, which includes the entire community of microbes living in the plant rhizosphere, phyllosphere and endosphere [9, 10], has received much attention for its potential to promote the health and productivity of agricultural crops [11]. For example, some plant-associated microbes can promote plant growth by fixing atmospheric nitrogen, solubilizing phosphorous, and producing siderophores that sequester iron, thereby increasing nutrient availability for plants. Others can promote plant growth by producing enzymes such 1-aminocyclopropane-1-carboxylic acid (ACC) deaminase, which breaks down stress ethylene and helps plants tolerate abiotic stress [12, 13]. Finally, some plant-associated microbes can help plants withstand pathogen stress via several mechanisms including competition, antibiosis, mycoparasitism and induced systemic resistance [14–16]. Individual microbial taxa can contribute to one or more of these so-called ‘plant growth promoting properties’, depending on their life cycle and environmental conditions [17–19]. Consequently, identifying key factors that regulate the composition and activity of plant microbiomes has potential to improve crop productivity and reduce reliance on agrochemical inputs to manage nutrients and pests.

Because resident soil microbial community structure is generally the dominant factor shaping the composition of plant microbiomes [11, 20] crop management practices that alter soil microbial community structure are likely to be critical for realizing the benefits of plant microbiomes in agricultural systems [21–24]. Recent studies indicate that plant genotype can also play a role in shaping the composition of plant microbiomes [25–28]. Moreover, the presence of distinct microbiomes have been observed among crop cultivars that are resistant and susceptible to plant pathogens [29–31], indicating that these microbes could play a role in mediating plant diseases [32]. Because differences in microbiome composition among plant genotypes has been demonstrated to be heritable in many plant species including maize and Arabidopsis [27, 28, 33], it may someday be possible to integrate selection for beneficial plant microbial relationships into breeding programs, offering exciting new possibilities for developing cultivars with improved disease resistance [34]. Endophytes, which are microbes that spend at least part of their life cycle living inside plants without causing disease [35], could provide a good starting point for breeders to determine if this is possible, because plants appear to act as ‘gatekeepers’, allowing only a subset of microbes in the rhizosphere to enter [36–39].

While some endophytic microbes have been demonstrated to promote plant growth, the functional role of many taxa, as well as factors that promote their activity within plants, are still not well understood [37, 40], which currently prevents growers or breeders from being able to exploit these plant-microbial relationships. For example, some endophytes appear to be in a commensal relationship with plants, with the microbe gaining nutrients and shelter while the plant appears to receive no direct benefit [37]. Since these microbes could represent a cost to the plant resulting in reduced fitness [36], the reason that plants would permit the continued presence of these microbes is unclear. Consequently, some have suggested that endophytes are simply latent saprotrophs or pathogens, waiting for the plant to senesce or for conditions to become conducive for disease development to occur [37, 40], and like pathogens, these microbes have developed unique mechanisms that allow them to evade plant immune systems [41]. In contrast, others have suggested that plants tolerate the presence of endophytes because they benefit the plant once it becomes subject to some stress [39, 42]. For example, *Epichloë* species can help Tall Fescue (*Festuca arundinacea)* plants withstand herbivory [43], and others can prime plants for faster and more intense defense responses once they are attacked by a pathogen [44]. As a result, some endophytes are vertically transmitted with seed [43], and could be part of a core microbiome [41], which has withstood domestication and breeding programs that have dramatically altered modern crop plants [45].

Despite the potential importance of endophytes in carrots, to our knowledge, only two studies have yet been conducted to investigate these plant-microbial relationships in this crop [46, 47], and while both demonstrated the potential for soil management to affect endophyte composition, neither investigated the potential functional role that endophytes could play during carrot production. Consequently, the primary goal of this study was to determine whether endophytes can help carrot plants withstand assault by *A*. *dauci*, a key pathogen in carrot production systems. In addition, we aimed to determine the extent to which crop management system and carrot genotype could interact to affect the potential for endophytes to help carrot plants tolerate this critical pest. Because if genotype does play a role, then it might be possible to select for varieties that support beneficial endophytes in carrot breeding programs to help better manage Alternaria blight.

## Material and methods

### Field trial

Carrot taproots were grown in a long-term crop systems trial comparing organic (ORG) and conventional (CNV) management at Purdue’s Meigs Horticultural Research Farm (lat. 40°17’21” N. long. 86°53’02”), located approximately 10 miles south of Lafayette, IN during summer 2014. Soil at this site is classified in the Drummer soil series, which typically contain approximately 3.2% organic matter and a neutral pH. The mean annual precipitation at this site is 1008 mm, and summer temperatures range from 21.1 to 26.7 °C. The crop systems trial was established in 2011 on adjacent tracts of land with uniform topography that had previously been managed using either organic or conventional farming practices since 2001. The crop systems trial was arranged in a split-block design, in which main plots represented the management system and subplots represented 36 experimental and commercial carrot genotypes, and there were three replicates for each management system X carrot genotype combination. Within each crop system, four cash crops, carrot, tomato (*Solanum lycopersicum*), popcorn (*Zea mays everta*) and soybean (*Glycine max*), were grown annually and managed using standard practices for each system. This included application of inorganic fertilizers and synthetic pesticides in the conventional system and inclusion of a winter cover crop and organic fertilizers in the organic system. The winter cover crop planted in the organic system consisted of a custom fall green manure mix containing winter rye (*Secale cereale L*.), hairy vetch (*Vicia villosa*), winter pea (*Pisum sativum*), annual rye (*Lolium perenne*), and timothy grass (*Phleum pratense*) (Cloverland Seed, Millersburg, OH). Cash crops were rotated in both crop systems annually in the following order: tomato -> carrot -> popcorn -> soybean.

In the carrot plots, fertilizers were applied to both systems to achieve a target rate of 134.5, 180 and 224 kg ha^-1^ of N, P and K respectively. In the organic plots, this consisted of Re-vita Pro Compost (Ohio Earth Foods, Hartville, OH), applied at a rate of 5,380 kg ha^-1^ to meet fertility needs, assuming 50% of the nutrients would be available for plant uptake in the year of application. In the conventional plots, diammonium phosphate (18-46-0) and potash (0-0-60) were applied to meet fertility needs. Sub-plots containing 36 carrot genotypes, which represented advanced breeding lines as well as commercial check cultivars, were randomized within each larger carrot plot. Untreated carrot seeds provided by Dr. Philipp Simon, USDA-ARS Vegetable Crop Research Unit, Madison, WI, were planted in mid-May. Seeds were planted on raised beds that were 1.8 m apart, in 1 m rows to provide approximately 60 plants m^-1^ per sub-plot given previously determined germination rates. Seeds were sown to a depth of 1 cm. In the conventionally managed system, a pre-emergent herbicide (Prowl H2O, BASF Corporation) was applied immediately after planting. In the organically managed system, plots were hand weeded as needed. No additional pesticides were applied in either crop management system. 110 days after seeding, the percentage of infection by foliar pathogens (including *Cercospora carotae*, *Alternaria dauci and Xanthomonas Xanthomonas campestris pv*. *Carotae*) in each plot was quantified using the Horsfall-Barratt rating scale [46]. In brief, each plot was assigned a numerical value from 1 to 12 corresponding to the percentage of leaf area showing leaf blight symptoms in each plot using the arbitrary Horsfall-Barratt rating scale in which 1 = 0% infection and 12 = 100% infection. Carrots were then manually harvested and the total number and weight of all taproots and aboveground foliage in each plot was recorded.

### Field soil chemical and biological assays

Ten soil cores were randomly collected to a depth of 10 cm in each field rep just prior to carrot seeding in spring. The ten cores within each field rep were pooled and transferred to the laboratory on ice. After thoroughly mixing the soil cores from each replicate, a subsample was air-dried before shipping to Midwest Labs (Omaha, NE) for a standard soil test according to common methods used in this region [47]. Briefly, total organic matter was determined using loss of weight on ignition; available P was extracted as Weak Bray (readily available P) and Strong Bray (potentially available P) and analyzed calorimetrically; exchangeable potassium (K), calcium (Ca), and magnesium (Mg) were extracted with neutral ammonium acetate (1 N) and quantified by inductively coupled argon plasma–mass spectrometry detection; and base saturation and cation exchange capacity [mmol (+)·kg–1] were estimated from the results of exchangeable minerals [47]. Another subsample was placed in the cooler at 4 °C until being air-dried overnight to conduct assays to estimate microbial activity and active soil carbon. Microbial activity was estimated using the hydrolysis of fluorescein diacetate (FDA) in soil slurries using a method optimized for soil [48]. Active C was quantified using the permanganate oxidizable carbon (POXC) technique [49]. Finally, a subsample was lyophilized and stored at -20, before being shipped overnight on dry ice to WARD lab (Grand Island, NE) for phospholipid fatty acid analysis (PLFA) using methods described in [50].

### Isolation and enumeration of culturable endophytes in carrot taproots

At harvest (110 days after seeding), nine of the 36 carrot genotypes planted in the larger trial were selected for use in this study based on their country of origin, differences in top size and tap root color/shape, and resistance to pathogenic soil nematodes and *A*. *dauci* (Table 1). Ratings for *A*. *dauci* and root knot nematodes in this table were developed using methods described in [51] and [52], respectively, and represent at least five years of observations in breeding nurseries conducted annually in Wisconsin and California by the USDA-ARS Carrot Breeding Program led by Phillip Simon. Two healthy carrot taproots were collected from each of the three sub-plots in each crop management system, for a total of six taproots for each of the nine genotypes evaluated in this trial. Taproots were collected from healthy plants with no symptoms of foliar or root diseases, or any other signs of plant stress. All carrot taproots were placed in a cooler on ice and transferred to the lab where they were stored at 4° C until processing within 48 hours. Isolation of endophytes in the taproots was conducted using methods previously described by [53]. Briefly, carrot taproots were rinsed very well with tap water before being surface disinfected by soaking taproots in 5.25% bleach for 3 minutes, followed by soaking in 3% peroxide solution for 3 minutes, and finally washing with sterilized water supplied with 1ml of tween [54]. To confirm surface disinfection of the carrot taproots, 200 μl samples from the last washing solution were plated onto various semi-selective media described below for broad microbial groups. Carrot cores were also rolled over the surface of each semi-selective media. All of these plates were incubated at 27 °C for 14 days and evaluated daily for the growth of microbial colonies.

**Table 1:** Carrot genotypes grown in conventional and organically managed systems at Purdue’s Meigs Farm during summer 2014.

Classification	Genotype description	Origin	Taproot color (external/internal)	Tap root shape	Nematode Gall Ratings	Alternaria leaf blight Rating
*Experimental breeding lines with novel root colors and tall tops for weed competitiveness*						
Experimental	Exp P6306	Turkey/Europe	Purple/Yellow	Imperator	6 (susceptible)	4–5
Experimental	Exp Y8519	Turkey/Europe	Yellow/Yellow	Imperator	7 (susceptible)	2.5–3.5
Experimental	Exp PY0191	Asia	Purple/Purple	Imperator	7 (susceptible)	4–5
Experimental	Exp B0252	Syria	Purple/Orange	Imperator	2 (moderately resistant)	4–5
*Nematode resistant breeding lines with high beta-carotene*						
Experimental	Exp Nb3999	Brazil/Europe	Orange/Orange	Imperator	1–2 (resistant)	3.5–4
*Open-pollinated populations with nematode resistance and tall tops for weed competitiveness*						
Commercial	Brasilia	Brazil	Orange/orange	Nantes	2–3 (moderately resistant)	2–3
Commercial	Nantes Scarlet Fancy Favorite (NSFF)	Europe	Orange/orange	Nantes	1–2 (resistant)	4–5
*Standard open-pollinated populations with tall tops for weed competitiveness*						
Commercial	Karotan	Europe	Orange/Orange	Flakee	5–6 (susceptible)	3–4
Commercial	Red Core Chantenay (RCC)	Europe	Orange/Orange	Chantenay	6–7 (susceptible)	2.5–3.5
*Current U*.*S*. *hybrid cultivars*						
Commercial	Napoli	United States	Dark orange/Dark Orange	Nantes	6–8 (susceptible)	3–5

Five cores were collected from each surface sterilized carrot taproot using a sterile 15 mm cylinder core borer. Each core was collected from mid-root and include both xylem (core) and phloem tissues. After mixing the cores collected from each plot, 5 grams of the cores were ground in 25 ml sterile water using an Omni tissue master homogenizer (OMNI International, GA., United States) to create a stock solution. The stock solutions were serially diluted ten times, and 100 *μ*l of each dilution was spread onto plates containing selective media for heterotrophic bacteria (Tryptic Soy Agar), oligotrophic bacteria (R2A), and total fungi (1/5th PDA media) [51, 55], each with two replicates. The petri plates were incubated at 27 °C or 25 °C and counted after 48 or 72 hours, for bacterial and fungal enumeration respectively.

Plates with serial dilutions of 10³ or 10^4^ and 10^4^ or 10^5^ were used to isolate individual fungal and bacterial colonies respectively, with unique morphologies. Each individual microbial isolate was inoculated onto a clean petri plate and incubated at 27 °C or 25 °C for fungi and bacteria respectively, to facilitate growth. The hyphael tip technique was used to further purify fungal cultures [56], and the streak plate technique was used to further purify bacterial cultures with agar slants [57]. Individual bacterial and fungal cultures that were morphologically distinct were selected and stored in glycerol stocks at -80C for future DNA extraction and laboratory assays.

### Identification of endophytes

Individual cultures of endophytic microbes stored at -80C were revived by culturing on fresh PDA or Luria-Bertani Agar media in petri plates before subjecting cultures to DNA extraction. DNA extraction was conducted using Microbial DNA extraction kits (Mo Bio, Laboratories, C.A., U.S.A) following the manufacturers recommendations. The final concentration and quality of DNA from each isolate was quantified using a nanodrop (Thermo Scientific^™^ NanoDrop^™^ 2000/2000c Spectrophotometers, U.S.A), before being diluted to l ng using Promega nuclease free water. For amplification of fungi, the universal ITS5 forward (5 ′ GGAAGTAAAAGTCGTAACAAGG- 3′) and ITS4 reverse (5′-TCC TCC GCT TAT TGA TAT GC- 3′) primers were used to amplify the whole ITS region [58]. Each 25-μl PCR reaction mixture contained 2μl of DNA template, 0.5 μl of each primer (100 mM), 12.5 μl GoTaq^®^ colorless Master Mix from Promega and 9.5 μl Promega nuclease free water. PCR reactions were performed in a Bio-Rad T100 thermal cycler (BioRad, C.A, U.S.A) using the following cycle conditions: initial denaturing step of 1 cycle at 95°C for 2 minutes, 40 cycles of (denaturing step: 95°C for 30 seconds, annealing step: 49°C for 30 seconds, extension step: 72°C for 1 minute), and a final extension step of 72°C for 10 minutes. Amplification of DNA from bacterial cultures was conducted using the 8F universal forward (5′-AGAGTTTGATCCTGGCTCAG- 3′) and 1492R universal reverse (5′- GGTTACCTTGTTACGACTT- 3′) primers [59], to amplify the V1-V9 (full length) hypervariable region of the 16S SSU rRNA gene of bacteria. The 25-μl PCR reaction mixture contained 1μl of DNA template, 0.5 μl of each primer (100 mM), 12.5 μl GoTaq^®^ colorless Master Mix from (Promega, WI. U.S.A) and 10.5 μl Promega nuclease free water. PCR reactions were performed in a Bio-Rad T100 thermal cycler using the following cycle conditions: initial denaturing step of 1 cycle at 94°C for 3 minutes, 35 cycles of (denaturing step: 94°C for 45 seconds, annealing step: 50°C for 60 seconds, extension step: 72°C for 90 seconds) and a final extension step of 72°C for 10 minutes. Verification of PCR-amplification was performed by electrophoresis on a 0.7% (wt./vol.) agarose gel stained with Bullseye DNA Safe Stain (MIDSCI., MO. U.S.A). A 100bp ladder (New England bio lab, MA. U.S.A) was run in parallel with the PCR products on each gel to approximate product band size. Presence of DNA bands stained with DNA Safe Stain were visualized after exposure of the gel to ultraviolet (UV) light. Amplified PCR products were cleaned using Ultra Clean ^®^ PCR Clean-Up Kits (MO BIO Laboratories, Laboratories, C.A., U.S.A), before being sent to the Purdue Genomics Facility for sequencing using an ABI 3137XL capillary machine (ABI company, CA., U.S.A) using forward primers.

Nucleotide sequences obtained through Sanger sequencing were analyzed using the Basic Local Alignment Search Tool (BLAST) [60], of the National Institutes of Health GenBank database [61], for precise identification of bacterial endophytes, and identification of fungal nucleotide sequences were further confirmed using the UNITE Genome Database [62]. Consistent with other studies, a 98% confidence level cut off was used for identification of bacteria [63], and a 97% confidence level cut off was used for identification of fungi at the species level [64]. All sequences have been submitted to the NIH GenBank database to obtain accession numbers.

### In vitro screening of endophyte isolates for antagonistic activity against *Alternaria dauci*

Unique endophytic isolates obtained following sequencing, representing 22 bacteria and 6 fungi, were screened using an *in vitro* assay to quantify antagonistic activity against *A*. *dauci*. The *A*. *dauci* isolate used in this experiment was previously isolated from a local carrot field and identified using DNA extraction, PCR amplification and sequencing as described above. The pathogenicity of the *A*. *dauci* isolated was subsequently confirmed using Koch’s postulates under greenhouse conditions. To obtain working bacterial cultures, isolates stored in -80C glycerol stocks were streaked onto fresh LB plates (10 g tryptone, 5 g yeast extract, 10 g NaCl and 15 g/L agar in 950 mL deionized water) [51], and incubated at 27°C for 2 days before single colonies were selected for use in the antagonistic screening assay. To obtain fungal endophyte and *A*. *dauci* working cultures, 5mm diameter mycelial plugs from each fungal stock were transferred onto fresh PDA plates and incubated at 25° C for 7 days before use in the antagonistic screening assay. All antagonistic tests were conducted on petri plates filled with PDA media. To screen fungal endophytes, 5mm diameter mycelial plugs from both *A*. *dauci* and the fungal isolate were placed 4.5 cm away from each other on the same petri plate [65]. To screen bacterial endophytes, a 5 mm diameter disk of *A*. *dauci* was placed at the center of the PDA plate, and then the individual bacterial isolates where streaked 2.25 cm away from the *A*. *dauci* disk on both sides of the disk [66]. Plates containing only *A*. *dauci* pathogen disks served as a control. There were three replicate plates for each endophytic isolate as well as the control, and the entire experiment was repeated to confirm initial results. All plates were incubated at 25°C until the *A*. *dauci* culture in the control covered the entire plate, and then the diameter of the *A*. *dauci* colony in the control plates and plates containing the endophytic isolates were recorded using electronic calipers.

### Greenhouse trial to quantify the potential of select endophytic isolates to affect germination, seedling growth and tolerance to *Alternaria dauci* stress

Five bacterial isolates were selected based on their antagonistic activity towards *A*. *dauci* during the *in vitro* assay described above (Table 5). In addition, one treatment included a mixture of ten endophytic microbial isolates. Two *Pseudomonas fluorescence* isolates that have previously been shown to possess some of the most common and potent bacterial toxins responsible for plant protection against fungal pathogens in agricultural soils [67], were included as positive controls. This included *P*. *fluorescence* isolate Q2-87 (obtained from L. Thomashow, USDA-ARS Pullman, WA), which is known to produce 2,4-diacetylphloroglucinol (DAPG), and *P*. *fluorescence* CHA0 (obtained from the Culture Collection of Switzerland), which is known to produce pyrrolnitrin (PRN). To obtain working cultures, the bacterial isolates were streaked onto plates containing LBA media (10 g tryptone, 5 g yeast extract, 10 g NaCl and 15 g/L agar in 1000 mL deionized water) [51], and incubated for 24 hours at 26° C. A single bacterial colony from each isolate was used to inoculate sterile LB broth, and this was incubated overnight at 26° C on a rotary shaker set at 200 rpm to obtain cultures in the log phase of growth. All cultures were then diluted to equal concentrations, by adjusting their OD_600_ to 0.6.

Untreated seed of two popular commercial carrot cultivars (Red Core Chantenay and Napoli) that differ in morphological characteristics (ie. taproot shape, root:shoot ratio) and susceptibility to *A*. *dauci* (Table 1) were obtained from High Mowing Seed (VT, U.S.A) for use in the greenhouse trials. In addition, Napoli tends to have higher year to year variability in susceptibility to *A*. *dauci* than other varieties. Seeds were surface sterilized following methods described in [41], before being soaked in bacterial suspensions for 24 hours on a rotary shaker set at 200 rpm. Untreated carrot seeds were included as a control. All seeds were sown in sterile 5 x 25 cm deepots (Greenhouse megastore, IL., USA) filled with Fafard Potting Mix #1 (Sungro Horticulture, MA., U.S.A) that had previously been pasteurized by subjecting the potting mix to a temperature of 60° C for 72 hours. One carrot seed was sown in each deepot, and the pots then were kept in a mist chamber to facilitate germination. There were 20 replicates for all endophyte X genotype treatments. Germination was recorded 5 days post-planting, and percentage of seed germinated was calculated and used to compare treated and untreated carrot seed. Once all carrot seeds had germinated, seedlings were moved to a greenhouse set at 22 ° C during the day and 18° C during the night ± 1° C, with 50–70% relative humidity and 16 hours of daylight. The experiment was organized in a randomized complete block design with eight replicates to accommodate environmental variation within the greenhouse.

*A*. *dauci* inoculum preparation and carrot seedling inoculation was conducted using the same isolate described above. The inoculum was produced by placing disks of the virulent *A*. *dauci* isolate onto petri plates containing carrot leaf agar [68], and incubating plates in the dark for 10 days at 20°± 2 C, followed by a period of alternate exposure to 12 hours of dark and 12 hours of ultraviolet light for 10–15 days [68]. Petri plates containing *A*. *dauci* cultures were filled with sterile water and scraped with a sterile scalpel to dislodge *A*. *dauci* spores from the mycelium and obtain a spore suspension. The spore and mycelium suspension was filtered through two layers of cheesecloth to separate the conidial spores from the mycelium for use in the inoculation process. Finally, the spore suspension was adjusted to a concentration of 1 x 10 ^4^ spores per ml using a haemocytometer [69]. Leaves of carrot seedlings (~30-day old seedlings) were sprayed with equal amounts of the *A*. *dauci* spore suspension using an atomizer to deliver an equal amount of inoculum to each plant. Carrot seedlings in the control treatment were sprayed with sterile water supplemented with 0.05% of tween 20 (Sigma). Sixty days after planting, images of each plant were taken and used to quantify the percentage of infection by *A*. *dauci* using a disease scale described by [70, 71]. Carrot aboveground foliage (shoot) and taproot dry weight were recorded. The entire experiment was repeated three times to confirm results.

### Statistical analysis

All soil chemical properties, soil microbial biomass and activity, number of endophyte colony forming units obtained on each selective media, antagonistic activity of endophytic microbial isolates, percent germination of carrot seed, and dry weight were statistically analyzed using the general linear model procedure for ANOVA, and differences among treatment pairs were determined using the student’s t test at a p-value of 0.05, using the SAS JMP software package [72]. All data were checked for normality, homogeneity of variance and linearity prior to analysis, and were transformed when necessary.

## Results

### Impact of crop management system on soil chemical and biological properties, carrot foliar disease severity and yield in the field trial

Several chemical and biological soil properties differed between the two crop management systems evaluated. In particular soil pH, percent total organic matter, calcium and percent calcium on cation exchange sites were greater in the organic system, while percent hydrogen on cation exchange sites was greater in the conventional system (Table 2). Active soil organic matter estimated using the permanganate oxidizable carbon test did not differ between the two systems, though overall microbial activity estimated using the fluorescein diacetate enzyme assay was greater in the organic system (Table 2). Many components of the microbial biomass in these soils including total microbes, total bacteria, actinomycetes, gram negative bacteria, total fungi, arbuscular mycorrhizal fungi, saprotrophs, and protozoa were greater in the organic than conventionally managed production system (Table 3). In addition, the predator:prey ratio was greater in the organic system, indicating that soils in the organic systems were healthier and could have greater pathogen suppressive activity.

**Table 2:** Soil chemical properties and microbial activity in carrot field managed using organic and conventional farm practices just prior to planting in summer 2014 at Purdue’s Meigs Horticulture Research Farm.

Crop System	%OM	P- weak bray	P—strong bray	K	Mg	Ca	pH	CEC	%K	%Mg	%Ca	%H	POXC	FDA
		Ppm							percent base saturation				mg POXC/kg soil	ug FDA/g soil/h
CNV	2.6 b^z^	139.7	151.3	488.3	314.3	1840.0 b	5.6 b	17.1	7.3	15.3	53.7 b	23.6 a	300.5	30.3 b
ORG	3.1 a	38.3	85.7	298.3	349.7	2190.0 a	6.4 a	15.7	4.8	18.5	69.6 a	7.1 b	330.8	49.8 a

^z^Different letters within a column represent significant difference as determined by Tukey’s honestly significant difference test (P < 0.05). %OM: percent total organic matter; POXC: permanganate oxidizable carbon; FDA: hydrolysis of fluorescein diacetate; CEC: Cation exchange capacity.

**Table 3:** Biomass of microbial groups in soils managed using organic and conventional management just prior to planting during summer 2014 at Purdue’s Meigs Horticulture Research Farm.

a)	Microbial Biomass (PLFA)										
System	Total Biomass	Total Bacteria	Actinomycetes	Gram (+) Bacteria	Gram (-) Bacteria	Rhizobia	Total Fungi	Arbuscular Mycorrhizal Fungi	Saprophytes	Protozoa	Undifferentiated
CNV	1266.1 b^z^	704.2 b	140.4 b	474.1	230.1 b	6.4	87.6 b	13.3 b	74.3 b	0.0 b	807.6
ORG	2223.7 a	1219.9 a	259.9 a	795.0	424.9 a	0.0	203.4 a	68.7 a	134.7 a	8.6 a	791.8
b)	Ratios of microbial biomass groups										
System	Fungi:Bacteria	Predator:Prey	Gram (+): Gram (-)	Sat:Unsat							
CNV	0.133	0 (all prey) b	2.348	2.684							
ORG	0.167	0.007 a	1.931	1.816							

^z^Different letters within a column represent significant difference as determined by Tukey’s honestly significant difference test (P < 0.05).

**Fig 1:**
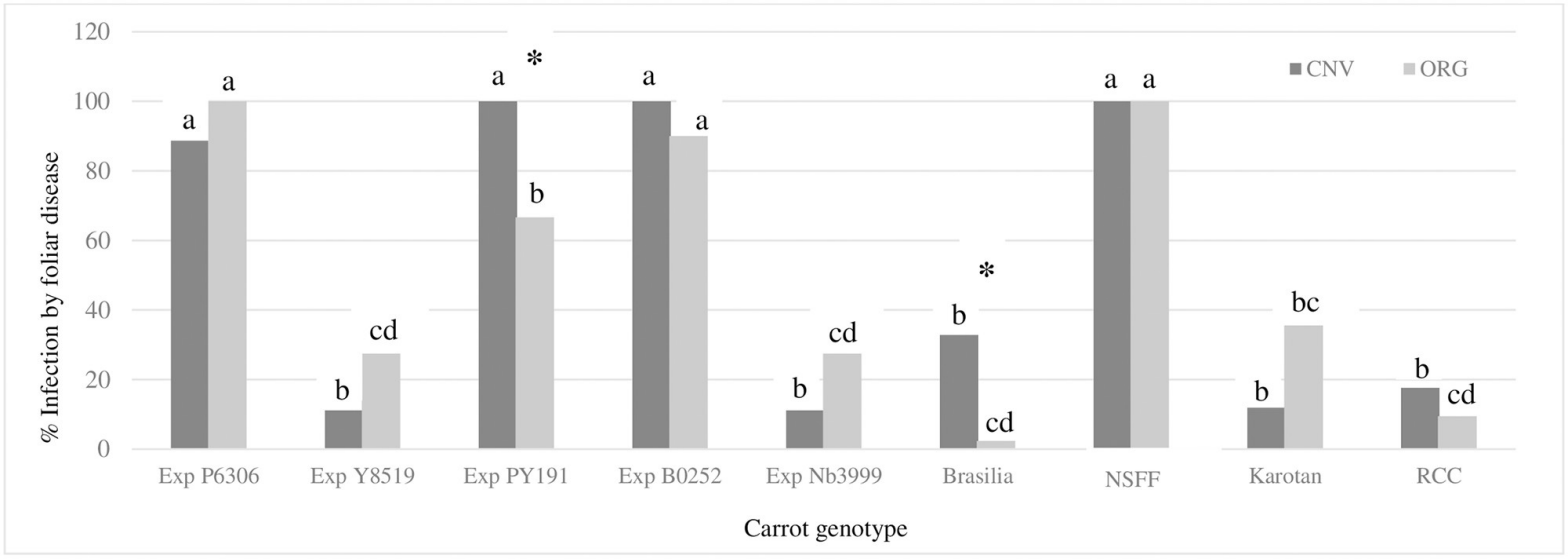
Percentage of carrot aboveground foliage displaying leaf blight disease symptoms including Alternaria in ten diverse carrot genotypes grown under organic and conventional management at Purdue’s Meigs Farm during summer 2014. Letters indicate significant differences between genotypes within each management system, and a star represents differences in management system within individual genotypes (*P*<0.001).

The total number of plants and weight of aboveground foliage and taproots of the carrot genotypes did not differ between the two crop management systems (S1 Table). The total percentage of aboveground carrot foliage with disease symptoms differed among the nine genotypes with Exp Y8519, Brasilia, Karotan,RCC and Napoli exhibiting relatively low rates in comparison to Exp P6306, Exp PY191, Exp B0252, E3999 and NSFF (Fig 1). Based on results of previous assays to identify carrot pathogens present in both of our organic and conventional fields ((91); L. du Toit personnel communication), we expect that disease symptoms in the aboveground foliage collected in this trial were likely caused by *Alternaria dauci Cercospora carotae* and *Xanthomonas campestris*. Two of the nine carrot genotypes (Exp PY191 and Brasilia), had significantly lower rates of foliar disease severity when grown in the organic compared to the conventional management system, but there were no statistical differences between systems in the other genotypes (Fig 1).

### Impact of crop management system and carrot genotype on the density of culturable endophytes in carrot taproots

**Fig 2:**
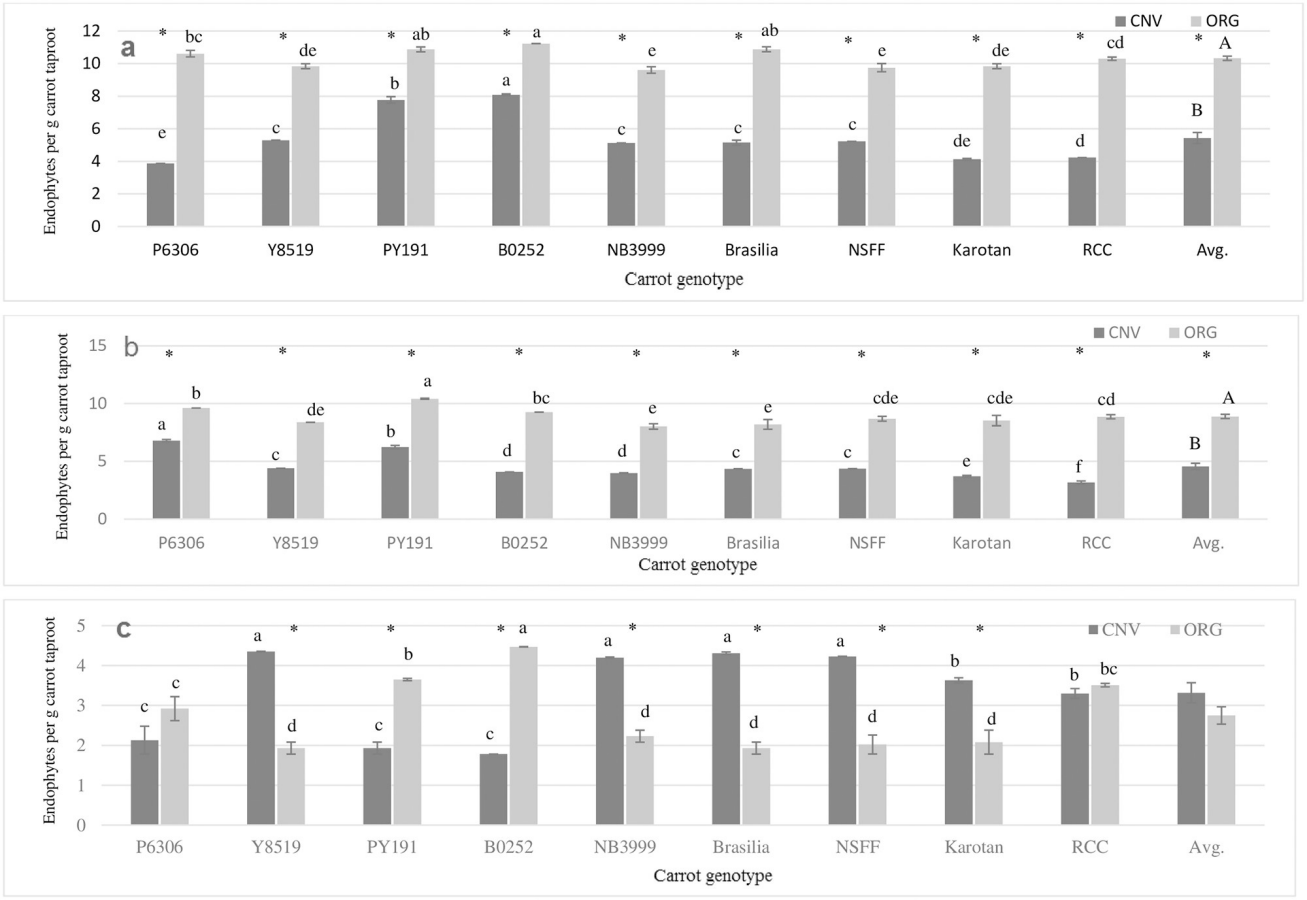
Abundance of heterotrophic bacteria (a), oligotrophic bacteria (a), and total fungi (c) isolated from the taproots of nine diverse carrot genotypes grown under organic and conventional management. Letters indicate significant differences between genotypes within each management system, and a star represents differences in management system within individual genotypes (*P*<0.001).

An abundant and diverse assortment of bacterial and fungal endophytes were isolated from the taproots of the nine diverse carrot genotypes grown under organic and conventional management in this trial (Fig 2 and Table 4). Heterotrophic bacteria, which are broadly defined as microbes that can respond quickly to the presence of labile carbon substrates, were the most abundant group of endophytic microbes isolated from carrot taproots regardless of management system or carrot genotype (Fig 2). Oligotrophic bacteria, which are broadly defined as slower growing bacteria that can survive in low nutrient conditions, were the second most abundant microbial group isolated in this study. When averaged across carrot genotype, the abundance of both heterotrophic and oligotrophic bacteria was significantly greater in carrot taproots grown under the organic management system (Fig 2). While not as dramatic as crop management, there were also differences in heterotroph and oligotrophic bacterial abundance among genotypes within both crop management systems (Fig 2). In some cases, individual carrot genotypes had the greatest or lowest abundance of these broad bacterial groups regardless of the crop management system in which they were grown. For example, PY191 and B0252 had high abundance and Kartotan had low abundance of heterotrophic bacteria in both systems. Among oligotrophic bacteria, P6306 and PY191 had high abundance in both systems, while many of the other genotypes had lower abundance of this broad bacterial group.

**Table 4:** Identification of unique endophytic bacteria (a) and fungi (b) recovered from the taproots of nine diverse carrot genotypes grown in field soil managed using organic and conventional farming practices during summer 2014 at Purdue’s Meigs Horticulture Research Farm.

a)						
	Isolate code	*Closest strain on NCBI data base*	E value	Identity	NCBI Accession # of closest hit	NCBI deposit Accession #
ORG	OB1	*Pseudomonas* sp.	0	100%	KX953866.1	SAMN13006134
	OB2	*Stenotrophomonas* sp.	0	99.46%	CP037883.1	SAMN13006142
	OB3	*Pseudomonas* sp.	0	99%	KX758046.1	SAMN13006141
	OB4	*Stenotrophomonas* sp.	0	100%	MH465193.1	SAMN13006132
	OB5	*Stenotrophomonas* sp.	0	100%	MH465193.1	SAMN13006130
	OB6	*Pseudomonas* sp.	0	97%	CP015225.1	SAMN13006137
	OB7	*Xanthomonas* sp.	0	100%	KY446031.1	SAMN13006140
	OB8	*Rhizobium* sp.	0	99%	KP751382.1	SAMN13006138
	OB9	*Pseudomonas* sp.	0	99.54%	MK610450.1	SAMN13006135
	OB10	*Xanthomonas* sp.	0	100%	KM252981.1	SAMN13006129
	OB11	*Pseudomonas* sp.	0	100%	LC420206.1	SAMN13006134
	OB12	*Pseudomonas* sp.	0	100%	FJ225306.1	SAMN13006133
	OB13	*Paenibacillus* sp.	0	100%	HF954523.1	SAMN13006131
	OB14	*Methylobacterium* sp.	0	99%	FN868937.1	SAMN13006136
CNV	CB1	*Rhizobium* sp.	0	100%	KU947328.1	SAMN13006122
	CB2	*Stenotrophomonas* sp.	0	99%	MH465193.1	SAMN13006123
	CB3	*Bacillus* sp.	0	100%	MG593988.1	SAMN13006127
	CB4	*Xanthomonas* sp.	0	100%	KY446031.1	SAMN13006121
	CB5	*Xanthomonas* sp.	0	100%	KM252981.1	SAMN13006128
	CB6	*Xanthomonas* sp.	0	99%	MH470420.1	SAMN13006124
	CB7	*Bacillus* sp.	0	100%	KX570915.1	SAMN13006125
	CB8	*Xanthomonas* sp.	0	99%	JQ698512.1	SAMN13006126
b)						
Treatment	Isolate code	*Closest strain on NCBI data base*	E value	Identity	NCBI Accession # of closest hit	NCBI deposit Accession #
ORG	OF1	*Epicoccum* sp.	0	100%	MF972508.1	SAMN13006143
	OF2	*Plectosphaerella* sp.	0	100%	MG004766.1	SAMN13006146
	OF3	*Colletotrichum* sp.	0	99.82%	JX294041.1	SAMN13006144
	OF4	Uncultured fungus	0	100%	KF768338.1	SAMN13006145
CNV	CF1	*Cladosporium* sp.	0	100%	MF154612.1	SAMN13006147
ORG/CNV	O/CF1	*Cladosporium* sp.	0	99%	KX641947.1	SAMN13006148

*98% and 97% confidence level cutoff were used for bacterial and fungal sequences identification at species level respectively. The accession # reported reflects the most positive match with the blast database.

When comparing the abundance of fungal endophytes isolated from carrot taproots there was no difference between management systems across the nine genotypes, however, there was an interaction between crop management system and carrot genotype (Fig 2). For example, Y8519, NB3999, Brasilia, NSFF and Karotan had greater abundance of fungal endophytes in the conventional system, while PY191 and B0252 had greater abundance in the organic system.

### Identification of unique endophytic microbial taxa recovered from nine diverse carrot genotypes grown under organic and conventional management

A total of 36 unique microbes were isolated from surface sterilized carrot taproots based on morphological characteristics. Following amplification and sequencing of 16s rRNA and ITS variable regions, 28 distinct microbial species were identified; 22 of these were bacterial and 6 were fungi (Table 4). Based on search results in BLAST, the endophytic bacterial isolates were identified as belonging to *Pseudomonas*, *Xanthomonas*, *Stenotrophomonas*, *Rhizobium*, *Bacillus*, *Paenibacillus* and *Methylobacterium* genera, and fungal taxa were members of the *Cladosporium*, *Plectosphaerella*, *Colletotrichum* and *Epicoccum* genera, as well as one uncultured fungus that could not be positively identified. The top hit for the closest accession number in the BLAST system was the same for some isolates, though based on differences in morphological characteristics, antagonistic activity and other closest hits in the BLAST system, we expect that these isolates are distinct. Out of the 22 unique bacterial endophyte isolates recovered in this study, 14 were isolated from carrots grown under the organic management system, while only 8 were isolated from carrots grown under the conventional management system. Among fungal endophyte isolates, 6 unique taxa were recovered from carrot taproots grown under organic management, while only 2 were recovered from carrot taproots grown under conventional management.

### Impact of crop management system and genera on the antagonistic activity of endophytic isolates against *Alternaria dauci*

**Fig 3:**
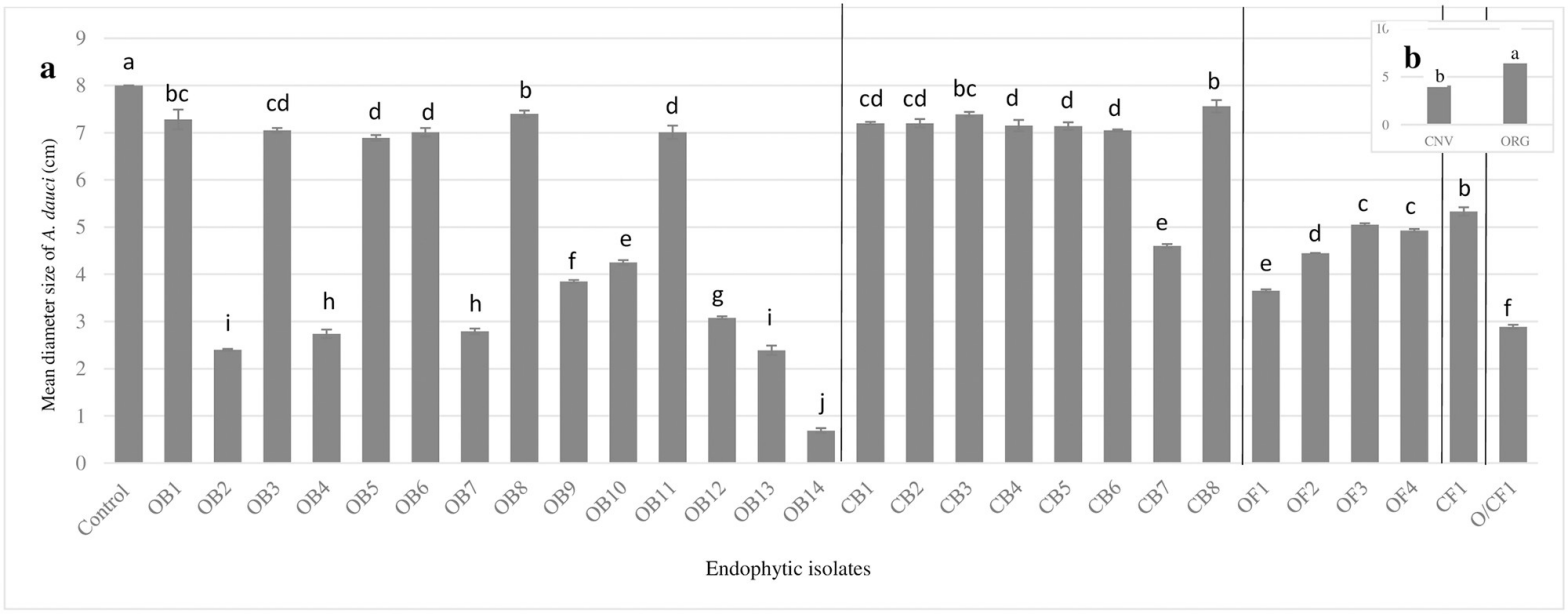
Antagonistic activity of individual endophytic microbes isolated from the taproots of nine diverse carrot genotypes grown in soil managed using organic and conventional farming practices (a) and endophytic isolates averaged across management system (b) against *Alternaria dauci* using an in vitro bioassay. Letters indicate significant differences between the endophytic isolates and management system (*P*<0.001).

All of the 22 bacterial and 6 fungal endophytes isolated from carrot taproots demonstrated some capacity to reduce the growth of *A*. *dauci* compared to the control during *in vitro* assays (Fig 3). When all isolates were considered, bacterial and fungal endophytes obtained from carrot taproots grown under organic management had greater antagonistic activity than those grown under conventional management (Fig 3B). Distinct differences in antagonistic activity among individual isolates collected from carrot taproots were also observed (Fig 3A). Bacteria isolated from taproots grown in the organic system with the greatest antagonistic activity against *A*. *dauci* included those belonging to genera of *Stenotrophomonas* (OB2, OB4), *Xanthomonas* (OB7, OB10), *Pseudomonas* (OB9, OB12), *Paenibacillus* (OB13) and *Methylobacterium* OB14. Only one isolate obtained from carrot taproots grown in the conventional system, which was identified as belonging to the *Bacillus* genera (CB7), had a relatively high degree of antagonistic activity against *A*. *dauci*. Fungi isolated from carrot taproots also had antagonistic activity, but were less effective than the bacterial isolates (Fig 3A).

### Potential for select endophytic isolates to affect germination, early plant growth and stress caused by *A*. *dauci* in two popular carrot cultivars

**Fig 4:**
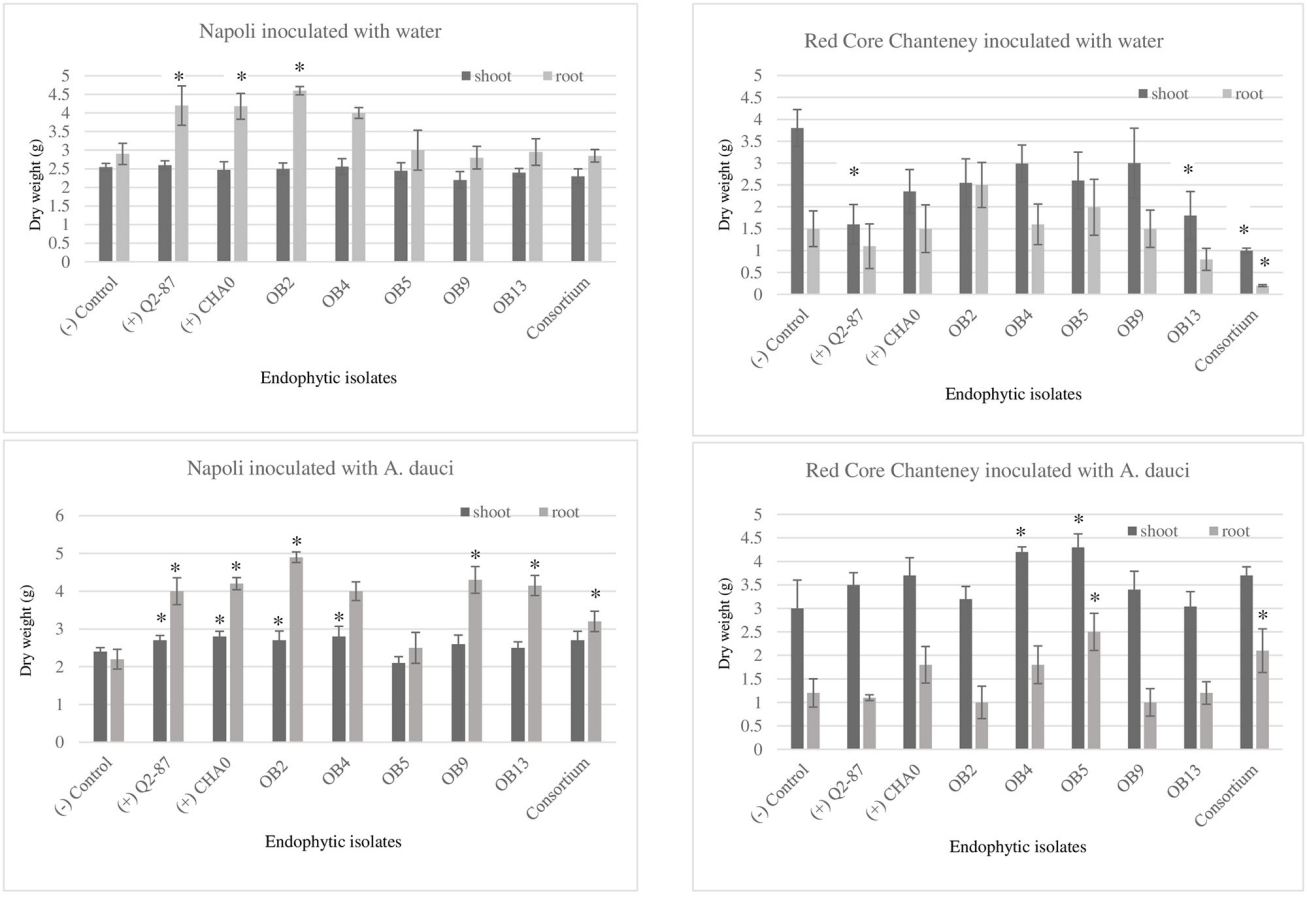
Shoot and root weight of two carrot varieties treated with endophytic bacterial isolates as a seed treatment and inoculated with water or *A*. *dauci* in a greenhouse trial. Stars indicate significant differences between the endophyte treatment and the negative control within each genotype by pathogen treatment and shoot and root category (*P*<0.001).

The two carrot genotypes selected for the greenhouse trials responded differently to inoculation with the endophyte isolates collected from carrot taproots grown in the field trial. For example, there were no significant differences in germination between endophyte-treated and untreated carrot seed of cv. Red Core Chantenay, whereas germination of cv. Napoli was reduced by the application of OB5, OB9 and OB13 (Table 5). In contrast, in the absence of *A*. *dauci*, seed treatment with Q2-87 and OB13 reduced shoot growth and the microbial consortium reduced both root and shoot growth in cv. Red Core Chantenay, whereas Q2-87, CHA0, OB2 and OB4 increased root growth in cv. Napoli (Fig 4). When carrot plants were inoculated with *A*. *dauci*, seed treatment with Q2-87, CHA0 and OB2 increased both root and shoot growth, OB4 increased shoot growth, and OB9, OB13 and the microbial consortium increased shoot growth in cv. Napoli (Fig 4). When cv. Red Core Chantenay plants were inoculated with *A*. *dauci*, OB5 increased both shoot and root growth, OB4 increased shoot growth, and the consortium increased root growth relative to the negative control (Fig 4). Only one of the positive control treatments (Q2-87) significantly reduced *A*. *dauci* disease symptoms, and none of the treatments significantly reduced *A*. *dauci* disease symptoms in Red Core Chantenay (S1 Fig).

**Table 5:** The effect of seed treatment with endophytic bacterial isolated from carrot taproots on the % germination of two carrot varieties in greenhouse trials.

Endophyte treatment	Isolate identify	Carrot genotype	
		Napoli	Red Core Chantenay
Negative control		85 ± 8.19 ab^z^	40 ± 11.24 a
Positive control (Q2-87)	*Pseudomonas fluorescens*	65 ± 10.94 abc	30 ± 10.51 a
Positive control (CHA0)	*Pseudomonas fluorescens*	90 ± 6.88 a	45 ± 11.41 a
OB2	*Stenotrophomonas* sp.	60 ± 11.24 bcd	35 ± 10.94 a
OB4	*Stenotrophomonas* sp.	65 ± 10.94 abc	25 ± 9.93 a
OB5	*Stenotrophomonas* sp.	30 ± 10.51 e	45 ± 11.41 a
OB9	*Pseudomonas* sp.	35 ± 10.94 de	31.58 ± 10.96 a
OB13	*Paenibacillus* sp.	55 ± 11.41 cde	30 ± 10.51a
Consortium (OB2, OB4, OB5, OB7, OB8, OB9, OB10, OB11, OB13)	*Stenotrophomonas sp*., *Stenotrophomonas sp*., *Stenotrophomonas sp*., *Xanthomonas sp*. *Pseudomonas sp*., *sp*., *Xanthomonas sp*, *Pseudomonas sp*., *Pseudomonas sp*., *Paenibacillus sp*. *Xanthomonas sp*.	65 ± 10.94 abc	50 ± 11.47 a

^z^Different letters within a column represent significant difference as determined by Tukey’s honestly significant difference test (P < 0.05).

## Discussion

Endophytic microbes have potential to help carrot plants plant withstand a wide range of biotic and abiotic stresses, resulting in improved crop performance, and reducing the need to rely on agrochemicals to manage production challenges. However, the identity and potential functional role of endophytes in carrot roots is still unclear, which prevents carrot growers from being able to leverage these communities to enhance crop performance. Consequently, the primary goal of this study was to identify endophytic microbes residing within carrot taproots, and determine if they can help carrot plants withstand stress by *A*. *dauci*, one of the most destructive pathogens in carrot production systems [2, 7, 73]. Secondly, we aimed to determine how crop management system and carrot genotype could interact to affect the potential for carrot plants to host endophytes with potential to help mediate *A*. *stress*.

Like previous studies [54, 74], carrot taproots in our trial were colonized by an abundant (Fig 2) and diverse assortment of endophytic microbes (Table 4) We recovered 28 distinct taxa (Table 4), though we suspect that the total diversity of endophytes present in carrot taproots could be even higher. For example, other studies have isolated between 92 to 1000 bacterial and 75 to 350 fungal species in the roots of other plant species [75–82]. Consequently, we expect that by using alternative techniques such as next generation sequencing, additional endophytic taxa will be identified in carrot taproots. Based on *in vitro* assays, we determined that all of the endophytes isolated in this trial have at least some capacity to reduce the growth of a virulent isolate of *A*. *dauci* previously collected from our field site (Fig 3). This not surprising given that most endophytes have been demonstrated to provide at least some level of antagonistic activity against pathogens [83, 84]. However, the suppressive activity of individual endophytic taxa varied by as much as 300%, indicating that some have greater potential to suppress disease caused by *A*. *dauci*, while others may provide additional benefits to carrot plants.

Bacteria isolated from carrot taproots belonged to *Proteobacteria*, *Firmicutes* and Bacteriodes phyla (Table 4), which is consistent other studies demonstrating that bacterial endophytes are generally dominated by a few phyla: Proteobacteria (∼50% in relative abundance), Actinobacteria (∼10%), Firmicutes (∼10%) and Bacteroidetes (∼10%) [85]. Endophytes must possess specialized features to move towards roots, enter and evade plant immune systems [39, 86], therefore it is not surprising that a select group of taxa often represents the majority of microbes present in this component of the plant microbiome. It is unclear why we did not isolate any Actinobacteria in this trial, though it could be due to the fact that these bacteria are not well suited to survive inside carrot taproots, or they may not grow as well on the selective media used to isolate the endophytes in this trial. At the level of genera, bacterial endophytes represented *Pseudomonas*, *Xanthomonas*, *Stenotrophomonas*, *Rhizobium*, *Bacillus*, *Paenibacillus* and *Methylobacterium* (Table 4). Many of these genera have been collected from other economically important plants including carrot [54, 87, 88], providing further evidence that they are common as endophytes and appear to have a broad host range. *Rhizobium*, *Bacillus*, *Stenotrophomonas* and *Pseudomonas* genera have been demonstrated to provide plant growth promoting properties, as well as antagonistic activity against a number of plant and human pathogens in many studies [54, 87, 88], indicating that they are likely to benefit carrot plants. Some *Xanthomonas* and *Pseudomonas* genera have been implicated for their potential to act as pathogens in several crops including carrot [4, 89], though this is not always the case. For example, [90] isolated *Xanthomonas* sp. from healthy rice seeds and determined that they could promote the health of rice plants by improving germination under salt stress. Similarly, endophytic microbes belonging to several *Pseudomonas* genera were isolated from healthy grass plants grown under nutrient-poor sand dunes, and found to possess the ability to fix atmospheric nitrogen under these conditions [91]. Consequently, in addition to helping carrot plants tolerate *A*. *dauci* stress, these endophytes could also provide additional benefits to carrots crops.

Fungi isolated from carrot taproots belonged to *Cladosporium*, *Plectosphaerella*, *Colletotrichum* and *Epicoccum* genera (Table 4). The fungus *Epicoccum nigrum*, has been deployed as a biological control agent against plant pathogens [92], providing further evidence that this isolate might be able to help carrots withstand stress by pathogens such as *A*. *dauci*. In addition, this particular microbe could possibly contribute to carrot nutritional quality. For example, [93] reported that *Epicoccum nigrum* can produce four types of carotenoid pigments. Carrot taproots are well known for their potential to be an important source of carotenoids in the human diet. Consequently, it would be interesting to determine whether the presence of this endophyte could indeed alter these compounds in carrot taproots. *Cladosporium*, *Colletotrichum* and *Plectosphaerella*, have been implicated as potential plant pathogens in several studies [94–96], supporting the assumption that they could be latent pathogens waiting for conditions to become conducive for disease development in carrot plants. However, *C*. *tofieldiae*, *C*. *oxysporum*, *C*. *sphaerospermum* and *P*. *cucumerina* endophytes have also been isolated from healthy pine (*Pinus* sp.) and soapberry (*Sapindus saponaria*) trees, as well as Arabidopsis (*Arabidopsis thaliana*) and sugar cane (*Saccharum officinarum*) plants [21, 97–100], demonstrating that they do not always cause disease. Moreover, some of the these microbes were able to produce bioactive products such as antibiotics and other anti-fungal compounds that were antagonistic to a number of plant and human pathogens [21, 97–100], indicating that they can play a positive role in plant as well as human health. Finally, the presence of *C*. *tofieldiae* has been shown to help Arabidopsis plants deal with phosphorous stress [101], providing further evidence that these microbes can indeed be beneficial for plants. Consequently, we suspect that the fungal isolates identified in this trial were not pathogens. However, we cannot rule out the possibility that they could contribute to disease outbreaks under the right conditions, as plant microbiomes can play a role in preventing, as well as exacerbating plant diseases [40].

As expected, crop management system altered the composition of endophytes (Table 4), which is consistent with previous reports in carrot [54, 74]. In particular, endophyte communities were more abundant and diverse in carrot taproots grown in the organic management system (Fig 2; Table 4), and individual isolates collected from this system had greater antagonistic activity towards *A*. *dauci* (Fig 3), indicating that soils in the organic system could have greater ‘disease suppressive potential’. Other studies have observed greater potential for disease suppressive activity in organic relative to conventional farming systems, which were thought to be related to changes in soil microbial community structure induced by different management practices [102–106]. For example, [104] reported that using synthetic fertilizer in a conventional system significantly reduced microbial biomass as well as abundance of *Trichoderma* and several thermophilic bacterial species, which have been noted for their role in pathogen suppression. Soils in the conventional system in that trial also had greater densities of pathogenic *Phytophthora* and *Pythium* species, providing further evidence that management induced changes in soil health can play important roles in pathogen dynamics. According to [107], greater suppressive activity in organic relative to conventional crop management systems is due to higher inputs of organic materials in organic systems, which support greater soil microbial biomass and diversity, leading to greater competition for resources, and hence greater antagonistic activity against pathogens. Greater populations of endophytic bacteria present in plant roots in organic systems like ours, also have potential enhance suppressive activity. This is because many bacterial processes such as disease suppressive activity, are regulated by fluctuations in cell-population density in a process known as quorum sensing [108]. For example, quorum sensing by the biological control agent *Serratia plymuthica* HRO-C48, was critical for the suppression of Verticillium in oilseed rape [109].

Since soils represent the microbial ‘seed bank’ where most plant microbiomes are recruited from [110], we suspect that greater concentrations of soil microbial biomass and activity in the organic soils in our trial (Table 3), likely contributed to the differences in endophyte community structure observed. Many previous studies have demonstrated that changes in soil properties resulting from different crop management practices can affect the composition of soil and plant microbiomes [21, 23, 24]. For example, [111] recovered 239 unique endophyte isolates from tomato, corn, melon and potato grown in an organic management system that had greater soil health, whereas only 97 were recovered from the same vegetables grown under conventional management. In our trial, we suspect that differences in soil properties we observed were due to repeated applications of organic fertilizers, the integration of winter cover crops, and the exclusion of chemical pesticides used in the organic system. Amending soils with organic fertilizers and incorporating cover crops provides organic substrates needed to support soil microbial biomass, diversity and activity in intensively managed agricultural systems [23, 67, 112]. These practices can also alter the structure of soil microbiomes indirectly, by changing soil physical and chemical soil properties such as aeration and pH [22, 113–115]. Finally, chemical herbicides and pesticides can reduce soil microbial biomass via non-target effects on a wide range of soil organisms [116]. Other factors that could have contributed to differences in endophyte communities observed in this trial, were management-induced changes in plant physiological status [40, 83]. For example, changes in plant nutrient status [117] and presence of pathogens [118], have been shown to alter the composition of endophyte communities in plants. We did observe significantly lower soil pH in the conventional system (Table 2), which could have altered the availability of nutrients and indirectly affected the composition of endophytes in carrot taproots. In contrast, we did not observe differences in the type of pathogens present in the two systems and taproots were collected from healthy plants, so we do expect that pathogens affected endophyte composition in the carrot taproots in our trial.

Developing cultivars that are resistant to plant pathogens has long been one of the most effective ways to prevent disease outbreaks in agricultural systems. In this trial, we observed distinct differences in foliar disease severity among the nine genotypes evaluated, providing evidence that some of these genotypes have some degree of resistance to foliar pathogens that include *A*. *dauci* (Fig 1). Moreover, because we observed differences in foliar disease severity between the management systems in two of the carrot genotypes, it is plausible that some aspect of the organic system, such as endophyte community composition, was helping to mediate disease symptoms in two these genotypes. Interestingly, unlike what we observed for bacteria, the total density of fungal endophytes in the taproots of all carrot genotypes grown in the organic system were not greater (Fig 2), despite the fact that like bacteria, there was also a greater abundance of total fungal biomass in soils from the organic system (Table 3). Instead, we observed an interaction between carrot genotype and crop management system, which could indicate that fungal endophytes in carrot taproots are more affected by plant genetic factors such as differences in root characteristics and plant physiology, than soil resident microbial community structure alone. Other studies have provided evidence plant genotypes can harbor distinct plant microbiomes [25, 29, 31, 119–122], and this in turn can have important implications for plant health and productivity. For example, [123] observed differences in plant growth among three potato cultivars inoculated with *Paenibacillus* spp and *Methylobacterium mesophilicum* endophytes. Similarly, modern and wild rice cultivars responded differently to inoculation with diazotrophic bacteria that can fix atmospheric nitrogen, which appeared to be related to differences in rooting behavior and composition of root exudates between the cultivars [124]. Based on the methods used to isolate endophytes in this trial, we cannot confirm if there were distinct differences in endophyte communities between the carrots genotypes that could have contributed to the differences in foliar disease severity observed, though this possibility should be explored in future trials.

To confirm that the endophytes isolated in this trial have potential to help carrot plants tolerate *A*. *dauci* stress, and determine the extent to which carrot genotype can play a role in this beneficial plant-microbial relationship, we inoculated two popular commercial cultivars of carrot (Napoli and Red Core Chantenay) with select endophytes isolated from our field trial. We chose these cultivars because they vary in morphological characteristics (ie. root size and root:shoot ratio), as well as in susceptibility to *A*. *dauci* (Table 1). Interestingly, the two carrot genotypes did differ in germination, early plant growth, and tolerance to stress caused by the presence of *A*. *dauci* when treated with the endophytes (Table 5; Fig 4), confirming that genotype can play an important role in regulating these plant-microbial relationships. Specifically, none of the endophyte treatments affected germination in Red Core Chantenay, whereas several reduced germination in Napoli. This indicates that some of these endophytes may have been acting as potential pathogens in Napoli, and may have even been outcompeting beneficial endophytic microbes that were vertically transmitted with the seed of this genotype. In contrast, we observed an opposite effect with respect to early seedling growth, as four of the treatments increased root growth in Napoli and three of the treatments reduced shoot growth in Red Core Chantenay (Table 5; Fig 4). Moreover, the beneficial effects of the endophytes on plant growth were even greater in Napoli with the presence of the pathogen *A*. *dauci*, with most treatments increasing root and shoot growth compared to the control. Only one of the positive control treatments significantly reduced *A*. *dauci* disease symptoms (S1 Fig), though reduction in disease symptoms in response to the other endophyte treatments were correlated with increases in plant growth (Fig 4), providing further evidence that these microbes can help Napoli withstand *A*. *dauci* stress. In contrast, the negative growth effects of the endophytes in Red Core Chantenay were no longer apparent with the presence of *A*. *dauci*, but only three of the treatments increased growth relative to the control (Fig 4), and none significantly reduced *A*. *dauci* disease symptoms (S1 Fig). These results further highlight the importance of context in regulating endophyte activities within plants [39, 40, 85, 125]. In addition, they demonstrate that while endophytes can represent a cost in some situations, they might be worth the cost in the long run, because they can benefit the plant once it becomes subject to some stress. The reason that Napoli generally responded more favorably to the inoculant treatments in these trials is unclear, though it could be due to the fact that this variety tends to invest more in its roots relative to shoots in comparison to Red Core Chantenay. Consequently, stimulation of root growth in response to endophyte inoculation might have been more dramatic in Napoli. In addition, the severity of *A*. *dauci* in this particular genotype is often more variable in year to year breeding trials than other genotypes (Table 1) indicating that endophyte communities might play a role in these differences.

Because of the strong potential for some endophytic taxa to promote plant growth and help suppress diseases, many isolates are being developed for use as inoculants in agricultural systems. Some of the endophytes isolated in our greenhouse trials such as OB2, improved carrot growth in the presence of the pathogen *A*. *dauci* as much as *P*. *fluorescence* Q2-87, a well-studied biocontrol isolate (Fig 4), indicating that OB2 may have potential for development as an inoculant to improve carrot productivity. However, because BLAST results indicated that this particular endophyte is likely to be a *Stenotrophomonas* sp., which can promote plant growth as well as act as an opportunistic pathogens in humans [126], the isolate would need to be subjected to a rigorous risk assessment before it could be deployed in agricultural systems [127]. While recent studies have demonstrated that some microbial taxa can work synergistically to enhance plant health, and thus inoculants containing a consortium of multiple microbial taxa could be more beneficial than single inoculants [128], this was not the case in our trial (Fig 4). This highlights the complexity that can occur within microbial consortiums, and that many additional studies will be needed to identify combinations of individual microbial taxa that can best help plants.

## Conclusions and potential implications of this research

Like most crops, carrot taproots are colonized by an abundant and diverse assortment of endophytic microbes. Some of these endophytes can directly suppress *A*. *dauci* and improve the productivity of carrot plants in the presence of this pathogen, providing evidence that these microbes can improve the health and productivity of carrot crops in the field. Moreover, it may be possible to increase the abundance of these endophytes in carrot taproots to aid in resistance to pathogens like *A*. *dauci*, by implementing soil-building practices commonly used in organic farming systems. Because the field trial in this study was only conducted for one year and growing season can affect the composition of plant endophyte communities [129, 130], the results of our trial need to be interpreted with caution. Nevertheless, because many studies have demonstrated that soil-building management practices commonly used in organic farming systems can improve soil and plant health [23], carrot growers should seriously consider implementing these practices as they could help compensate for the need to use pesticides and improve the productivity of their crops. The results of our trial also confirm that the composition of endophyte communities in taproots can vary among carrot genotypes, and carrot genotypes do differ in their response to the presence of individual entophytic taxa that can reduce stress caused by *A*. *dauci*. This indicates that carrot breeders could potentially begin to select for these beneficial plant-microbial relationships, and this should be explored in future studies.

## Supporting information

S1 Fig Disease severity in carrot cv. Napoli (a) and cv. Red Core Chanteney (b) treated with endophytic bacterial isolates as a seed treatment and inoculated with *A*. *dauci* in a greenhouse trial. ^z^Different letters within a column represent significant difference as determined by Tukey’s honestly significant difference test (P < 0.05).
(DOCX)

S1 Table Total number of carrot plants, and above and below ground biomass of ten carrot genotypes grown under conventional and organic management grown during summer 2014 at Purdue’s Meigs Farm south of Lafayette, IN. (DOCX)
