# Supplementary material for: Crop management system and carrot genotype affect endophyte composition and Alternaria dauci suppression
Source: PLoS One. 2020 Jun 4;15(6):e0233783. doi: 10.1371/journal.pone.0233783 (PMC7272071; doi:10.1371/journal.pone.0233783)
Supplement: S1 Fig — zDifferent letters within a column represent significant difference as determined by Tukey’s honestly significant difference test (P < 0.05). (DOCX) [file pone.0233783.s001.docx]

**a**

Endophytic isolates

Disease severity percentage

Disease severity percentage

Endophytic isolates

**S1 Fig** Disease severity in carrot cv. Napoli (a) and cv. Red Core Chanteney (b) treated with endophytic bacterial isolates as a seed treatment and inoculated with *A. dauci* in a greenhouse trial. ^z^Different letters within a column represent significant difference as determined by Tukey’s honestly significant difference test (P < 0.05).
