# Supplementary material for: Crop management system and carrot genotype affect endophyte composition and Alternaria dauci suppression
Source: PLoS One. 2020 Jun 4;15(6):e0233783. doi: 10.1371/journal.pone.0233783 (PMC7272071; doi:10.1371/journal.pone.0233783)
Supplement: S1 Table — (DOCX) [file pone.0233783.s002.docx]

**S1 Table .** Total number of carrot plants, and above and below ground biomass of ten carrot genotypes grown under conventional and organic management grown during summer 2014 at Purdue’s Meigs Farm south of Lafayette, IN

|  | **# of plants** | | **Root weight (g)** | | **Shoot weight (g)** | |
| --- | --- | --- | --- | --- | --- | --- |
| **Genotype** | **Conventional** | **Organic** | **Conventional** | **Organic** | **Conventional** | **Organic** |
| Exp P6306 | 16.0 | 9.0 | 2.32 | 1.03 | 1.19 | 0.50 |
| Exp Y8519 | 19.7 | 20.0 | 2.74 | 1.55 | 1.28 | 1.08 |
| Exp PY0191 | 10.0 | 13.0 | 1.14 | 1.20 | 0.34 | 0.33 |
| Exp B0252 | 12.0 | 11.0 | 0.83 | 0.67 | 0.31 | 0.52 |
| Exp Nb3999 | 14.0 | 12.7 | 2.06 | 1.61 | 0.56 | 0.38 |
| Brasilia | 9.0 | 15.7 | 1.38 | 1.56 | 0.67 | 0.79 |
| NSFF | 9.7 | 15.7 | 1.21 | 1.15 | 0.66 | 0.44 |
| Karotan | 10.7 | 14.7 | 1.67 | 1.77 | 1.11 | 0.76 |
| RCC | 13.7 | 21.3 | 2.02 | 1.34 | 0.96 | 0.53 |
| Napoli | 12.7 | 8.7 | 1.74 | 1.41 | 0.42 | 0.22 |

^z^Different letters within a column represent significant difference as determined by Tukey’s honestly significant difference test (P < 0.05).
